# The long-term impact of childhood sexual assault on depression and self-reported mental and physical health

**DOI:** 10.3389/fpsyt.2024.1528914

**Published:** 2025-01-23

**Authors:** Oluwasegun Akinyemi, Temitope Ogundare, Mojisola Fasokun, Fadeke Ogunyankin, Nkemdirim Ugochukwu, Walia Ajisafe, Seun Ikugbayigbe, Oluebubechukwu Eze, Kakra Hughes, Miriam Michael

**Affiliations:** ^1^ College of Medicine, Howard University, Washington, DC, United States; ^2^ Chobanian & Avedisian School of Medicine, Boston University, Boston, MA, United States; ^3^ Epidemiology, University of Alabama at Birmingham, Birmingham, AL, United States; ^4^ Research Data Science and Analytics, Cook Children’s Health Care System, Fort Wort, TX, United States; ^5^ Biological Sciences, Eastern Illinois University, Charleston, IL, United States

**Keywords:** adverse childhood experiences, depression, childhood sexual assault, self-reported mental health, self-reported physical health, inverse probability weighting, BRFSS behavioral risk factor surveillance system

## Abstract

**Background:**

Childhood trauma, including sexual assault (CSA), is a known risk factor for adverse mental health outcomes. This study quantifies the impact of CSA on the likelihood of being diagnosed with depression in adulthood, as well as its influence on poor mental and physical health days.

**Methods:**

We analyzed data from the Behavioral Risk Factor Surveillance System (BRFSS) (2016–2023), comprising 321,106 respondents. The primary exposure was self-reported CSA, while the main outcomes were depression diagnosis, poor mental health days, and poor physical health days. Covariates included race, gender, marital status, employment, age, education, state, year, language spoken at home, metropolitan status, and urban residence. We employed Inverse Probability Weighting (IPW) to estimate the Average Treatment Effect (ATE), controlling for confounders and incorporating state and year fixed effects. Sampling weights ensured national representativeness, and robust standard errors accounted for clustering by state.

**Results:**

In a matched cohort of 15,150 individuals with CSA and 15,150 controls, the CSA group had an average age of 50.3 ± 16.3 years, with most being White (69.3%) and female (76.7%). CSA was significantly associated with an increased risk of depression diagnosis, with a 22.1 percentage-point increase for those with one CSA experience (ATE = 0.221, 95% CI: 0.192–0.250, p < 0.001) and a 24.4 percentage-point increase for those with multiple CSA experiences (ATE = 0.244, 95% CI: 0.222–0.266, p < 0.001). CSA also impacted mental health. Those with a single CSA exposure reported 2.8 more days of poor mental health per month (ATE = 2.829, 95% CI: 2.096–3.398, p < 0.001), while those with multiple exposures reported 4.2 more days (ATE = 4.175, 95% CI: 3.609–4.740, p < 0.001) compared to controls. Regarding physical health, individuals with one CSA exposure reported 1.5 additional poor physical health days (ATE = 1.538, 95% CI: 0.788–2.289), while those with multiple exposures experienced 2.6 additional days (ATE = 2.587, 95% CI: 1.941–3.232).

**Conclusion:**

This study provides robust evidence that CSA significantly increases the likelihood of depression in adulthood and leads to more poor mental and physical health days. The findings underscore the cumulative impact of multiple CSA exposures on health outcomes and emphasize the need for trauma-informed healthcare, early intervention, and public health strategies to mitigate the long-term consequences of CSA.

## Introduction

Childhood sexual assault (CSA) is a pervasive and deeply traumatic experience that has profound implications for individual and population health (1–3). Defined as any forced or coercive sexual act experienced during childhood, CSA disrupts normal developmental processes and inflicts lasting psychological and physical harm (3, 4). The prevalence of CSA varies globally, but studies indicate that a significant proportion of adults having experienced some form of sexual abuse during their childhood, underscoring its widespread impact (5, 6).

Extensive research has shown that survivors of CSA are at heightened risk for numerous adverse outcomes that persist in adulthood (2, 7, 8). Among the most significant of these is the increased likelihood of developing mental health disorders, including depression, anxiety, and post-traumatic stress disorder (PTSD) (4, 9). Depression, in particular, poses substantial public health challenges as it is a leading cause of disability worldwide, affecting millions and imposing significant economic and social burdens (10, 11). Key studies, such as those by Fergusson et al. and Felitti et al., have documented the robust association between CSA and long-term mental health problems, establishing CSA as a critical risk factor for depression later in life (12, 13).

The mechanisms underlying this relationship are complex and multifaceted, involving both biological and psychosocial pathways. CSA has been linked to dysregulation of the hypothalamic-pituitary-adrenal (HPA) axis, which plays a crucial role in stress response (3, 8, 9, 14, 15). inflammatory responses, and alterations in neurobiological development (3, 8, 15, 16). These changes can impair emotional regulation and increase susceptibility to mental health disorders. In addition, survivors often engage in maladaptive coping strategies, such as substance abuse or withdrawal from social interactions, further compounding the risk of depression and other mental health issues (3, 17).

The impact of CSA extends beyond individual health, influencing population-level health metrics and contributing to the overall burden on healthcare systems (18). Public health initiatives that address the long-term effects of childhood trauma (19–21) are therefore essential for improving mental health outcomes and promoting well-being. Despite increased awareness, gaps remain in our understanding of the full extent of CSA’s impact and the effectiveness of interventions aimed at mitigating its effects.

This study builds upon existing research by employing a rigorous analytical approach to investigate the relationship between CSA and depression diagnoses in a large, nationally representative cohort. Understanding this relationship is vital for informing policy, shaping trauma-informed care practices, and developing preventive measures to support survivors and reduce the long-term consequences of CSA.

## Methodology

### Study design and data source

This study utilized data from the Behavioral Risk Factor Surveillance System (BRFSS), covering the period from January 2016 to December 2023 (22, 23). The BRFSS is a nationally representative, cross-sectional survey that collects data on health-related risk behaviors, chronic health conditions, and preventive health practices among U.S. adults (24). A total of 321,479 respondents were included in the analysis, ensuring a robust sample for generalizability.

### Primary explanatory variable

The main exposure variable was self-reported childhood sexual assault (CSA). This was categorized as a trichotomous variable (once, more than once and/never). This categorization was determined based on participants’ responses to questions related to adverse childhood experiences. Specifically, the survey asked: “How often did anyone at least 5 years older than you or an adult, force you to have sex?” with six response options: *Never*, *Once*, *More than Once*, *Don’t know/not sure*, *Refused*, and *Not asked or missing*. For the analysis, this variable was recoded into a trichotomous format. Responses of “Never” were coded as 0, indicating no history of childhood sexual assault. Responses of “Once” were coded as 1, representing a single occurrence of childhood sexual assault. Finally, responses indicating that the assault occurred more than once were coded as 2.

Responses marked as *Don’t know/not sure*, *Refused*, or *Not asked or missing* were excluded from the analysis.

### Outcome variables

The outcome variable, self-reported depression, was derived from responses to the survey question: “(Ever told) (you had) a depressive disorder (including depression, major depression, dysthymia, or minor depression).” The original response options were categorized into five groups: *Yes*, *No*, *Don’t know/Not sure*, *Refused*, and *Not asked or Missing*. For analytical purposes, this was recoded into a binary variable indicating depression status. Responses of *No* were coded as 0 (no diagnosis of depression), while responses of *Yes* were coded as 1 (diagnosis of depression). Responses of *Don’t know/Not sure*, *Refused*, and *Not asked or Missing* were excluded from the analysis.

The final outcome variable represented self-reported diagnoses of depression, based on past or current medical assessments by healthcare professionals.

### Secondary analysis

This study employed a secondary analysis to assess the relationship between self-reported childhood sexual assault and individuals’ self-reported mental and physical health status within the past 30 days.

The mental health status was based on responses to the survey question: “Now thinking about your mental health, which includes stress, depression, and problems with emotions, for how many days during the past 30 days was your mental health not good?” Response options included: *Number of days*, *None*, *Don’t know/Not sure*, *Refused*, and *Not asked or missing*. For the purpose of analysis, the variable was operationalized as a continuous variable ranging from 0 to 30. Responses of *Don’t know/Not sure*, *Refused*, and *Not asked or missing* were excluded from the analysis.

The second secondary outcome, physical health status, captured responses to the survey question: “Now thinking about your physical health, which includes physical illness and injury, for how many days during the past 30 days was your physical health not good?” This variable had similar response categories: *Number of days*, *None*, *Don’t know/Not sure*, *Refused*, and *Not asked or missing*. For the purpose of analysis, the variable was operationalized as a continuous variable ranging from 0 to 30. Responses of *Don’t know/Not sure*, *Refused*, and *Not asked or missing* were excluded from the analysis.

#### Covariates and confounder adjustment

The analysis adjusted for a comprehensive set of potential confounders, including demographic and socio-economic factors: race/ethnicity, gender, marital status, employment status, age, and education level. Additional contextual variables included state of residence, year of data collection, primary language spoken at home, metropolitan status, and urban/rural classification. These covariates were included to account for known and potential confounding effects on both exposure and outcome.

### Statistical analysis

Inverse Probability Weighting (IPW) was employed to estimate the Average Treatment Effect (ATE) of childhood sexual assault on depression diagnosis. This method was chosen to mitigate selection bias by creating a weighted pseudo-population in which the distribution of covariates is independent of the exposure. The treatment model used a logistic regression framework to calculate propensity scores for each individual based on the covariates listed above. Sampling weights provided by BRFSS were applied to generate nationally representative estimates. To control for unobserved heterogeneity, state and year fixed effects were included in the model, addressing potential regional and temporal biases.

### Robustness and precision

Robust standard errors clustered at the state level were used to ensure the validity of the standard errors and confidence intervals, accounting for potential within-state correlation of responses.

#### Model estimation and interpretation

The primary analysis focused on estimating the ATE, representing the difference in the probability of depression diagnosis between individuals who experienced childhood sexual assault and those who did not. The model output included the ATE, potential outcome means (POmeans) for unexposed individuals, and 95% confidence intervals for all estimates. Statistical significance was determined at a p-value threshold of < 0.05.

#### Ethics and data access

The BRFSS dataset is publicly available and de-identified, ensuring that the study adhered to ethical standards for the use of human data without requiring additional Institutional Review Board (IRB) approval.

## Result

### Baseline characteristics

**Table 1** presents the baseline characteristics of 321,106 respondents, including 15,381 individuals with a history of childhood sexual assault (CSA) and 305,725 individuals without such exposure (unmatched sample). After propensity score matching, 15,150 individuals with a history of CSA were matched to 15,150 controls, substantially improving balance across key covariates.

**Table 1:** Baseline Covariate Balance for Unmatched and Matched Samples.

Covariates	Unmatched Sample (N=321,106)		% Bias	p-value	Matched Sample (N=30,300)		% Bias	p-value
	Control (n=305,725)	CSA (N=15,381)			Control (N=15,150)	CSA (15,150)		
Age (yrs)	56.6 ± 17.6	50.4 ± 17.0	-34.8	<0.001	50.8 ± 16.2	50.3 ± 16.3	-0.1	0.177
Female (ref. Male)	173667 (54.1%)	245312 (76.4%)	48.2	<0.001	11574 (76.4%)	11625 (76.7%)	-0.7	0.255
Race/Ethnicity								
White	250681 (78.1%)	222485 (69.3%)	-20	<0.001	10639 (70.2%)	10497 (69.3%)	-2.1	0.76
Black	30508 (9.5%)	40546 (12.6%)	10	<0.001	1913 (12.6%)	1963 (13.0%)	-1.1	0.217
Hispanic	20371 (6.3%)	27596 (8.6%)	8.6	<0.001	1302 (8.6%)	1214 (8.0%)	2.2	0.001
Other	13342 (4.2%)	16765 (5.2%)	5	<0.001	791 (5.2%)	752 (5.0%)	1.2	0.678
Multiracial	6204 (1.9%)	13714 (4.3%)	13.5	<0.001	647 (4.3%)	582 (3.8%)	2.5	0.343
Marital Status								
Married	170838 (53.2%)	128359 (40.0%)	-26.8	<0.001	6105 (40.3%)	6056 (40.0%)	-0.7	0.105
Single	51454 (16.0%)	60895 (19.0%)	7.7	<0.001	2873 (19.0%)	2890 (19.1%)	-0.3	0.705
Divorced	41124 (12.8%)	67506 (21.0%)	22	<0.001	3185 (21.0%)	3245 (21.4%)	-1.1	0.005
Widowed	41606 (13.0%)	31115 (9.7%)	-10.3	<0.001	1468 (9.7%)	1401 (9.2%)	1.4	0.001
Separated	5773 (1.8%)	15281 (4.8%)	16.7	<0.001	721 (4.8%)	709 (4.7%)	0.4	0.116
Member of an Unmarried Couple	10311 (3.2%)	17953 (5.6%)	11.6	<0.001	847 (5.6%)	800 (5.3%)	1.5	0.354
Employment Status								
Employed (ref. Unemployed)	155325 (48.4%)	147243 (45.9%)	-5	<0.001	6947 (45.9%)	7059 (46.6%)	-1.5	0.174
Education								
Less than High School	105686 (32.9%)	125687 (39.1%)	13	<0.001	5972 (39.4%)	5930 (39.1%)	-0.6	0.261
Some College	90411 (28.2%)	110618 (34.4%)	13.6	<0.001	5219 (34.4%)	5224 (34.5%)	-0.1	0.015
College Graduate	125010 (38.9%)	84801 (26.4%)	-26.9	<0.001	4001 (26.4%)	3954 (26.1%)	0.7	0.081
Metro (ref. non-metro)	217090 (67.6%)	216827 (67.5%)	-0.2	<0.001	10230 (67.5%)	10336 (68.2%)	-1.5	0.016
Urban (ref. Rural)	267828 (83.4%)	268923 (83.7%)	0.9	<0.001	12688 (83.7%)	12738 (84.1%)	-0.9	0.053
English (ref. Non-English Speaking)	314793 (98.0%)	314918 (98.1%)	0.3	<0.001	14858 (98.1%)	14884 (98.2%)	-1.2	0.029

In the unmatched sample, there were notable differences in key sociodemographic characteristics between individuals who reported CSA and the control group. The CSA group had a higher proportion of females compared to the control group (76.4% vs. 54.1%), with a large bias of 48.2%. The CSA group also had a lower proportion of married individuals (40.0% vs. 53.2%, bias: -26.8%) and college graduates (26.4% vs. 38.9%, bias: -26.9%). Racial and ethnic composition also differed, with the CSA group having higher proportions of Black, Hispanic, and multiracial individuals, contributing to biases ranging from 8.6% to 13.5%. Furthermore, the mean age was lower in the CSA group (50.4 ± 17.0 years) compared to the control group (56.6 ± 17.6 years), reflecting a bias of -34.8%.

After propensity score matching, the baseline differences between the CSA and control groups were substantially reduced, with all key covariates falling well within acceptable bias thresholds. The gender bias was reduced from 48.2% to -0.7%, and marital status biases were reduced to a range of -1.1% to 1.5%. Similarly, biases in educational attainment decreased to a range of -0.1% to 0.7%, and the mean age difference exhibited a minimal bias of -0.1%. Racial and ethnic distribution differences were also reduced, with biases for White, Black, Hispanic, and multiracial groups falling to values ranging from -2.1% to 2.5%.

These improvements indicate that propensity score matching successfully created a well-balanced sample of individuals with and without a history of childhood sexual assault, providing a robust basis for comparison of outcomes in the matched cohort (**Table 1**).

### Childhood sexual assault significantly increases risk of depression

This study examined the association between CSA and the likelihood of experiencing depression later in life (**Table 2**). The analysis revealed that CSA significantly increases the probability of being diagnosed with depression.

**Table 2:** Average Treatment Effects of Childhood Sexual Assault on Depression Incidence.

Outcome	CSA (Treated vs. Control)	Coef.	Std. Err.	z-value	p-value	95% CI	
Depression	(Once vs. Never)	0.221	0.015	14.810	0.000	0.192	0.250
	(More than once vs. Never)	0.244	0.011	21.690	0.000	0.222	0.266
	Never	0.185	0.001	134.400	0.000	0.182	0.188

This table presents the Average Treatment Effects (ATE) and predicted incidence of depression by treatment group. The coefficients (ATE) represent the average change in depression incidence relative to the “Never” group. z-values and p-values assess statistical significance, while the 95% confidence interval (CI) indicates the range where the true effect is expected to lie. Group comparisons are as follows: Individuals who experienced childhood sexual assault once are compared to those who never experienced it (“Once vs. Never”), individuals who experienced childhood sexual assault more than once are compared to those who never experienced it (“More than once vs. Never”), and the baseline predicted incidence of depression is reported for individuals with no history of childhood sexual assault (“Never”).

Individuals who experienced CSA once had a 0.221 (22.1%) higher likelihood of being diagnosed with depression compared to those who never experienced CSA (Coef = 0.221, SE = 0.015, z = 14.81, p < 0.001, 95% CI: 0.192 – 0.250). Similarly, individuals who experienced CSA more than once had a 0.244 (24.4%) greater likelihood of depression compared to those who never experienced CSA (Coef = 0.244, SE = 0.011, z = 21.69, p < 0.001, 95% CI: 0.222 – 0.266).

The predicted probability of depression among individuals who never experienced CSA was 0.185 (SE = 0.001, z = 134.40, p < 0.001), with a 95% confidence interval of 0.182 to 0.188. This suggests that approximately 18.5% of individuals with no history of CSA reported having been diagnosed with depression (**Table 2**).

### Childhood sexual assault on self-reported poor mental health days

**Table 3** presents the results of the analysis examining the association between CSA and the number of self-reported poor mental health days per month. The analysis estimates the ATE of CSA on self-reported poor mental health days, comparing individuals with a history of CSA (once or more than once) to those with no history of CSA.

**Table 3:** Average Treatment Effects of Childhood Sexual Assault on Self-Reported Poor Mental Health Days per Month.

Outcome	CSA (Treated vs. Control)	Coef.	Std. Err.	z-value	p-value	95% CI	
Mental Health	(Once vs. Never)	2.747	0.332	8.270	0.000	2.096	3.398
	(More than once vs. Never)	4.175	0.288	14.470	0.000	3.609	4.740
	Never	11.250	0.061	183.060	0.000	11.130	11.371

This table presents the Average Treatment Effects (ATE) and predicted number of days per month individuals reported having poor mental health, based on treatment group. The coefficients (ATE) represent the average change in the number of good mental health days relative to the “Never” group. z-values and p-values assess statistical significance, while the 95% confidence interval (CI) indicates the range where the true effect is expected to lie. Group comparisons are as follows: Individuals who experienced childhood sexual assault once are compared to those who never experienced it (“Once vs. Never”), individuals who experienced childhood sexual assault more than once are compared to those who never experienced it (“More than once vs. Never”), and the baseline predicted number of poor mental health days per month is reported for individuals with no history of childhood sexual assault (“Never”).

The results indicate that individuals who experienced CSA reported significantly more poor mental health days per month compared to those who never experienced CSA. Specifically, individuals with a history of CSA once reported an additional 2.747 poor mental health days per month compared to those with no history of CSA (Coef = 2.747, SE = 0.332, z = 8.27, p < 0.001, 95% CI: 2.096 to 3.398). Similarly, individuals who experienced CSA more than once reported an additional 4.175 poor mental health days per month relative to those with no CSA history (Coef = 4.175, SE = 0.288, z = 14.47, p < 0.001, 95% CI: 3.609 to 4.740).

The Potential Outcome Mean (POmean) for individuals with no history of CSA was 11.250 poor mental health days per month (SE = 0.061, z = 183.06, p < 0.001, 95% CI: 11.130 to 11.371). This indicates that, on average, individuals with no CSA history report approximately 11.25 poor mental health days per month.

These findings underscore a strong and statistically significant relationship between CSA and an increase in poor mental health days. The dose-response pattern observed, with higher exposure (more than one CSA experience) being associated with a greater number of poor mental health days, suggests that the frequency of CSA exposure plays a critical role in shaping mental health outcomes. The results highlight the cumulative impact of multiple CSA exposures on self-reported mental health (**Table 3**).

### Childhood sexual assault and self-reported poor physical health days

**Table 4** presents the results of the analysis examining the association between CSA and the number of self-reported poor physical health days per month. The analysis estimates the ATE of CSA on self-reported poor physical health days, comparing individuals with a history of CSA (once or more than once) to those with no history of CSA.

**Table 4:** Average Treatment Effects of Childhood Sexual Assault on Self-Reported Poor Physical Health Days per Month.

Outcome	CSA (Treated vs. Control)	Coef.	Std. Err.	z-value	p-value	95% CI	
Physical Health	(Once vs. Never)	1.538	0.383	4.020	0.000	0.788	2.289
	(More than once vs. Never)	2.587	0.329	7.860	0.000	1.941	3.232
	Never	11.040	0.066	168.440	0.000	10.911	11.168

This table presents the Average Treatment Effects (ATE) and predicted number of days per month individuals reported having poor physical health, based on treatment group. The coefficients (ATE) represent the average change in the number of good physical health days relative to the “Never” group. z-values and p-values assess statistical significance, while the 95% confidence interval (CI) indicates the range where the true effect is expected to lie. Group comparisons are as follows: Individuals who experienced childhood sexual assault once are compared to those who never experienced it (“Once vs. Never”), individuals who experienced childhood sexual assault more than once are compared to those who never experienced it (“More than once vs. Never”), and the baseline predicted number of poor physical health days per month is reported for individuals with no history of childhood sexual assault (“Never”).

The results indicate that individuals who experienced CSA reported significantly more poor physical health days per month compared to those who never experienced CSA. Specifically, individuals with a history of CSA once reported an additional 1.538 poor physical health days per month relative to those with no history of CSA (Coef = 1.538, SE = 0.383, z = 4.02, p < 0.001, 95% CI: 0.788 to 2.289). Similarly, individuals who experienced CSA more than once reported an additional 2.587 poor physical health days per month compared to those with no CSA history (Coef = 2.587, SE = 0.329, z = 7.86, p < 0.001, 95% CI: 1.941 to 3.232).

The potential outcome mean for individuals with no history of CSA was 11.040 poor physical health days per month (SE = 0.066, z = 168.44, p < 0.001, 95% CI: 10.911 to 11.168). This indicates that, on average, individuals with no CSA history report approximately 11.04 poor physical health days per month.

These findings reveal a significant association between CSA and an increase in poor physical health days. The observed dose-response pattern indicates that individuals with multiple CSA exposures experience more poor physical health days than those with a single exposure. These results highlight the cumulative impact of multiple CSA exposures on self-reported physical health outcomes (**Table 4**).

## Discussion

Our findings demonstrate a significant association between CSA and the development of depression later in life. This result is consistent with existing literature that highlights the strong link between early exposure to trauma and increased risk of mental health disorders (25–27). Studies such as those by Fergusson et al. (2013) and Hailes et al. (2019) have similarly reported that CSA is a substantial risk factor for developing depression and other affective disorders (18, 28). The probable causal link between CSA and depression involves complex pathways, including long-term alterations in stress response systems, such as dysregulation of the hypothalamic-pituitary-adrenal (HPA) axis, and the impact on emotional regulation and cognitive processing (8, 28, 29).

From a policy perspective, our findings underscore the need for comprehensive preventive measures, early intervention programs, and support systems aimed at mitigating the long-term psychological impacts of CSA. Public health strategies should incorporate screening for CSA in routine mental health evaluations and provide targeted therapeutic services for those affected. Our analysis found a significant association between CSA and self-reported poor mental health days aligning with previous research indicating that CSA is a powerful predictor of long-term mental health challenges (8, 28, 30). Studies such as those by Widom et al. (2007) and McLaughlin et al. (2010) have similarly shown that individuals who experienced CSA report higher levels of stress, anxiety, and emotional distress throughout their lives (31, 32).

The probable causal link between CSA and increased self-reported poor mental health days can be attributed to several interrelated factors. CSA often results in chronic activation of stress pathways, leading to dysregulation in the hypothalamic-pituitary-adrenal (HPA) axis, impaired emotional processing, and disrupted social functioning (3, 8, 33). These physiological and psychological effects can be manifested as prolonged periods of poor mental health, with survivors frequently facing difficulties in managing stress and emotional well-being.

The policy implications of these findings are profound. Public health initiatives should prioritize early identification and intervention for individuals exposed to CSA, integrating trauma-informed care within mental health services to mitigate its long-term effects. In addition, community-based prevention programs and educational campaigns that promote awareness and reduce stigma associated with reporting and discussing CSA are vital for fostering supportive environments (34–36).

Our study also reported a significant association between CSA and self-reported poor physical health days. This aligns with the existing literature, which highlights the long-term impacts of childhood trauma on physical health (37, 38). Research by Sachs-Ericsson et al. (2010) and Irish et al. (2010) has demonstrated that individuals who have experienced CSA are at an increased risk of chronic health conditions and report poorer general health outcomes compared to those without such experiences (7, 39).

The probable causal link between CSA and poorer physical health may involve several interconnected biological and psychosocial pathways. CSA is associated with chronic stress exposure, which can lead to dysregulation of the hypothalamic-pituitary-adrenal (HPA) axis and inflammatory responses (8, 16). Such dysregulation has been linked to the development of chronic physical health issues, including cardiovascular problems, musculoskeletal pain, and immune dysfunction (8, 40). In addition, survivors of CSA may engage in health-risk behaviors, such as substance use or poor dietary practices, as coping mechanisms, further exacerbating physical health issues (41–43).

The policy implications of these findings emphasize the necessity for trauma-informed healthcare practices that recognize and address the lasting effects of CSA on mental and physical health. Integrating routine screening for trauma history in primary care and ensuring accessible mental and physical health support services are critical steps. Public health initiatives should aim to promote early intervention and comprehensive care approaches that address both mental and physical health consequences of CSA.

### Strengths and Limitations

This study has several strengths that bolster its scientific rigor and reliability. By leveraging data from the Behavioral Risk Factor Surveillance System (BRFSS) over an eight-year period (2016–2023) and encompassing a large sample size of 321,106 respondents, our analysis achieves high statistical power and generalizability. The use of sampling weights ensures national representativeness, making the findings relevant to broader populations. Additionally, the application of Inverse Probability Weighting to estimate the Average Treatment Effect helps mitigate confounding bias, enhancing the robustness of our causal inferences. The inclusion of comprehensive covariates, as well as state and year fixed effects, provides control for potential regional and temporal variations, further strengthening the study’s methodological soundness.

Childhood sexual assault in this study was defined using responses to the survey question: “How often did anyone at least 5 years older than you or an adult, force you to have sex?”. This definition focuses on forced sexual encounters by older individuals or adults, effectively capturing the core aspect of coercive and non-consensual sexual acts in childhood. While this question is direct and ensures a clear understanding of what constitutes CSA, it may not fully encompass the breadth of childhood sexual abuse experiences, such as non-penetrative abuse, repeated psychological trauma, or more nuanced forms of coercion.

The strength of this definition lies in its specificity and ability to highlight severe cases of CSA, which are likely to have significant psychological and physical health impacts. By targeting forced sexual acts by individuals substantially older than the victim, it underscores a clear power imbalance, making it an essential measure for assessing severe CSA. However, its limitation is in its narrow focus; it may underreport cases of CSA that involve grooming, non-forced sexual acts, or perpetrators close in age to the victim. Additionally, it excludes experiences of abuse that may not be recognized as sexual assault by the respondents due to stigma or differing perceptions of abuse.

Other limitations that must be acknowledged in this study include the reliance on self-reported data for both the primary exposure (childhood sexual assault) and the outcome (depression diagnosis) introduces the potential for recall and reporting bias. The binary classification of depression does not capture the severity or spectrum of depressive disorders, potentially limiting the depth of interpretation. Moreover, while we adjusted for numerous covariates, residual confounding due to unmeasured variables, such as genetic predispositions or specific environmental factors, cannot be ruled out. The cross-sectional nature of the BRFSS data restricts the ability to establish temporal causality definitively, although robust statistical methods were employed to infer causality. Finally, while fixed effects control for regional and temporal variations, the findings may not fully account for cultural or socioeconomic differences across states that could influence the experience and reporting of CSA and mental health outcomes.

## Conclusion

This study provides compelling evidence of the long-term impact of childhood sexual assault on mental and physical health. Our findings demonstrate that CSA significantly increases the likelihood of depression in adulthood and is associated with a greater number of poor mental and physical health days. These results reinforce the well-established link between early trauma and lifelong health outcomes.

The pathways connecting CSA to poorer health outcomes are likely driven by biological and psychosocial mechanisms, including chronic stress, alterations in the hypothalamic-pituitary-adrenal (HPA) axis, and maladaptive coping behaviors. The cumulative impact of multiple CSA exposures further highlights the dose-response relationship between trauma severity and adverse health outcomes.

These findings underscore the urgent need for trauma-informed healthcare practices that incorporate routine screening, early intervention, and holistic support services. Interventions should aim to reduce the enduring mental and physical health burdens experienced by CSA survivors. Public health policies should prioritize preventive strategies and comprehensive trauma care to mitigate the long-term impact of CSA. Addressing these needs can enhance the overall well-being of CSA survivors, reduce healthcare costs, and alleviate the public health burden associated with childhood trauma.

## Data Availability

The raw data supporting the conclusions of this article will be made available by the authors, without undue reservation.
