# Supplementary material for: The long-term impact of childhood sexual assault on depression and self-reported mental and physical health
Source: Front Psychiatry. 2025 Jan 23;15:1528914. doi: 10.3389/fpsyt.2024.1528914 (PMC11799268; doi:10.3389/fpsyt.2024.1528914)
Supplement: Supplementary file 1 [file SupplementaryFile1.docx]

**Appendix 1**

**Flow Diagram**

Figure 1: Flow Diagram of Study Sample Selection

Control= 15,150

Treated (CSA)= 15,150

Missing: *N*

Depression+1630

English language:995

Education:995

Married:2318

Race:51,132

Employ:2,470

CSA= 321,106

Childhood sexual assault =380,646

Total Sample: 3,447,217

**Appendix 2**

**Sensitivity Analysis**

To address the potential influence of the **COVID-19 pandemic** on our study findings, we conducted a **sensitivity analysis** by excluding data from the pandemic years **2020, 2021, and 2023**. This approach allowed us to examine whether the observed associations between **Childhood Sexual Assault (CSA)** and key outcomes, including **incidence of depression**, **poor mental health days**, and **poor physical health days**, remained consistent in the absence of the potential confounding effects of the pandemic.

The COVID-19 pandemic is known to have disproportionately impacted mental and physical health, which could confound observed relationships. By excluding these years, we aimed to ensure that our findings were not driven by the broader health impacts of the pandemic.

Table 1: Average Treatment Effects of Childhood Sexual Assault on Depression Incidence

| Outcome | CSA (Treated vs. Control) | Coef. | Std. Err. | z | P | 95% CI | |
| --- | --- | --- | --- | --- | --- | --- | --- |
| Depression | (Once vs. Never) | 0.205 | 0.022 | 9.150 | < 0.001 | 0.161 | 0.249 |
|  | (More than once vs. Never) | 0.251 | 0.019 | 13.400 | <0.001 | 0.214 | 0.287 |
|  | Never | 0.194 | 0.002 | 82.050 | <0.001 | 0.189 | 0.198 |

This table presents the Average Treatment Effects (ATE) and predicted incidence of depression by treatment group. The coefficients (ATE) represent the average change in depression incidence relative to the "Never" group. z-values and p-values assess statistical significance, while the 95% confidence interval (CI) indicates the range where the true effect is expected to lie. Group comparisons are as follows: Individuals who experienced childhood sexual assault once are compared to those who never experienced it ("Once vs. Never"), individuals who experienced childhood sexual assault more than once are compared to those who never experienced it ("More than once vs. Never"), and the baseline predicted incidence of depression is reported for individuals with no history of childhood sexual assault ("Never").

Table 2: Average Treatment Effects of Childhood Sexual Assault on Self-Reported Poor Mental Health Days per Month

| Outcome | CSA (Treated vs. Control) | Coef. | Std. Err. | z | P>z | 95% CI | |
| --- | --- | --- | --- | --- | --- | --- | --- |
| Mental Health | (Once vs. Never) | 2.650 | 0.551 | 4.810 | <0.001 | 1.571 | 3.729 |
|  | (More than once vs. Never) | 4.120 | 0.494 | 8.340 | <0.001 | 3.151 | 5.088 |
|  | Never | 11.427 | 0.108 | 105.620 | <0.001 | 11.215 | 11.639 |

This table presents the Average Treatment Effects (ATE) and predicted number of days per month individuals reported having poor mental health, based on treatment group. The coefficients (ATE) represent the average change in the number of poor mental health days relative to the "Never" group. z-values and p-values assess statistical significance, while the 95% confidence interval (CI) indicates the range where the true effect is expected to lie.

Group comparisons are as follows: Individuals who experienced childhood sexual assault once are compared to those who never experienced it ("Once vs. Never"), individuals who experienced childhood sexual assault more than once are compared to those who never experienced it ("More than once vs. Never"), and the baseline predicted number of poor mental health days per month is reported for individuals with no history of childhood sexual assault ("Never").

Table 3: Average Treatment Effects of Childhood Sexual Assault on Self-Reported Poor Physical Health Days per Month

| Outcome | CSA (Treated vs. Control) | Coef. | Std. Err. | z | P>z | 95% CI | |
| --- | --- | --- | --- | --- | --- | --- | --- |
| Physical Health | (Once vs. Never) | 1.585 | 0.643 | 2.460 | 0.014 | 0.323 | 2.846 |
|  | (More than once vs. Never) | 1.887 | 0.505 | 3.740 | <0.001 | 0.897 | 2.877 |
|  | Never | 11.226 | 0.104 | 108.420 | <0.001 | 11.023 | 11.428 |

This table presents the Average Treatment Effects (ATE) and predicted number of days per month individuals reported having poor physical health, based on treatment group. The coefficients (ATE) represent the average change in the number of poor physical health days relative to the "Never" group. z-values and p-values assess statistical significance, while the 95% confidence interval (CI) indicates the range where the true effect is expected to lie. Group comparisons are as follows: Individuals who experienced childhood sexual assault once are compared to those who never experienced it ("Once vs. Never"), individuals who experienced childhood sexual assault more than once are compared to those who never experienced it ("More than once vs. Never"), and the baseline predicted number of poor physical health days per month is reported for individuals with no history of childhood sexual assault ("Never").
